# Supplementary figures and images for: Entia Non Sunt Multiplicanda … Shall I look for clusters in my cognitive data?
Source: PLoS One. 2022 Jun 30;17(6):e0269584. doi: 10.1371/journal.pone.0269584 (PMC9246139; doi:10.1371/journal.pone.0269584)

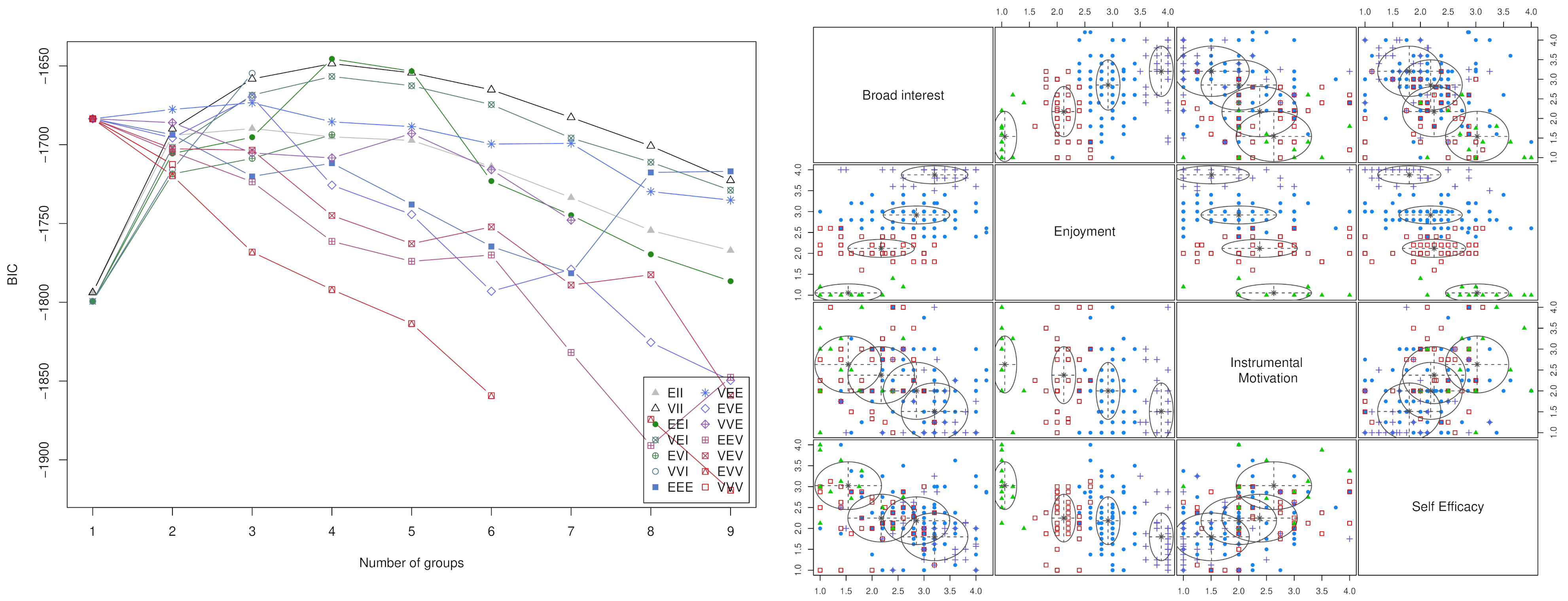

Supplement: S3 Fig — BIC values at the increasing number of latent groups for different structures of the variance-covariance matrix (left) and classification results obtained with 4 groups (right). (TIFF) [file pone.0269584.s003.tiff]

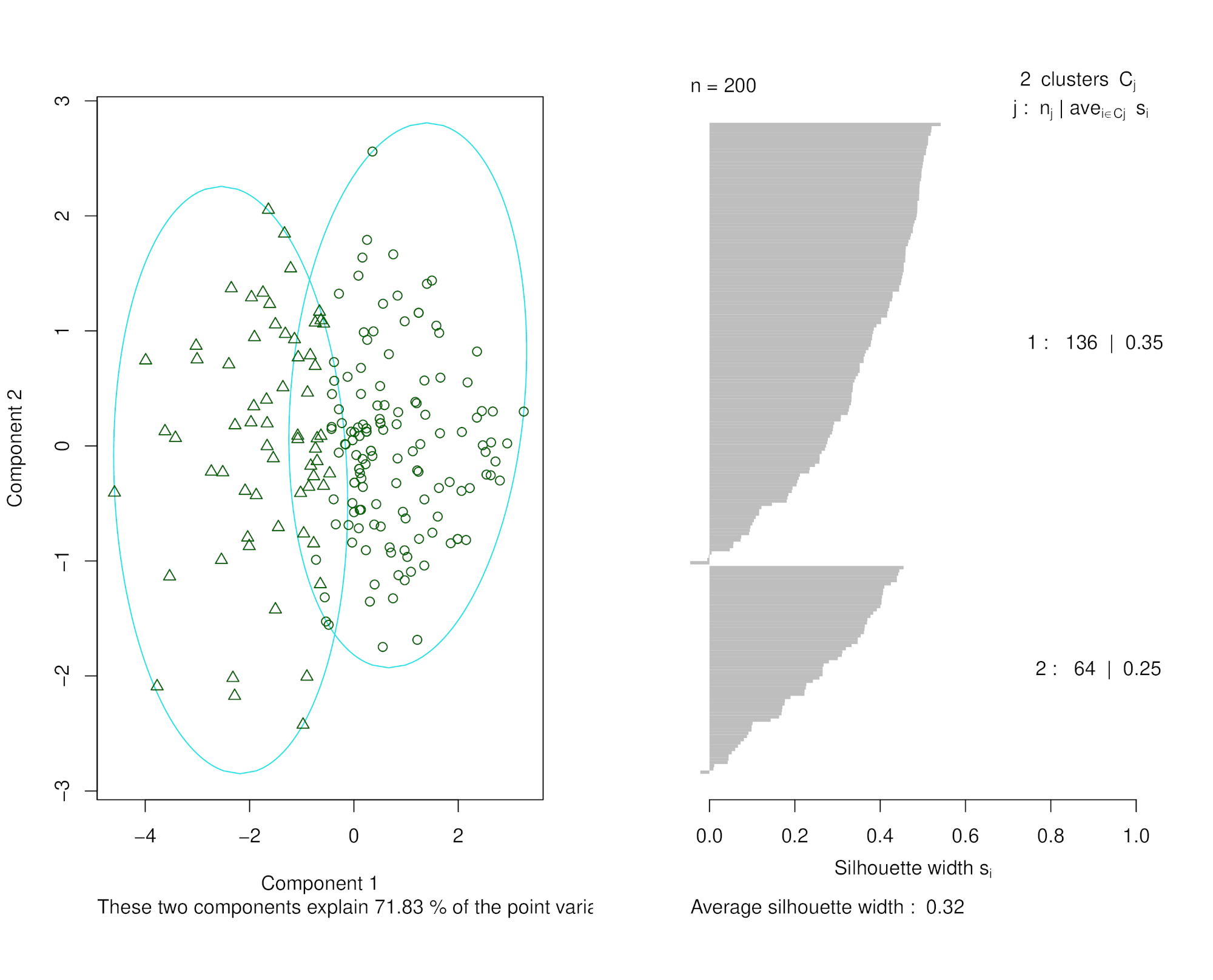

Supplement: S4 Fig — Clusters identified along two principal components (left) and silhouette width plot for the PAM clustering with 2 clusters (right). (TIFF) [file pone.0269584.s004.tiff]

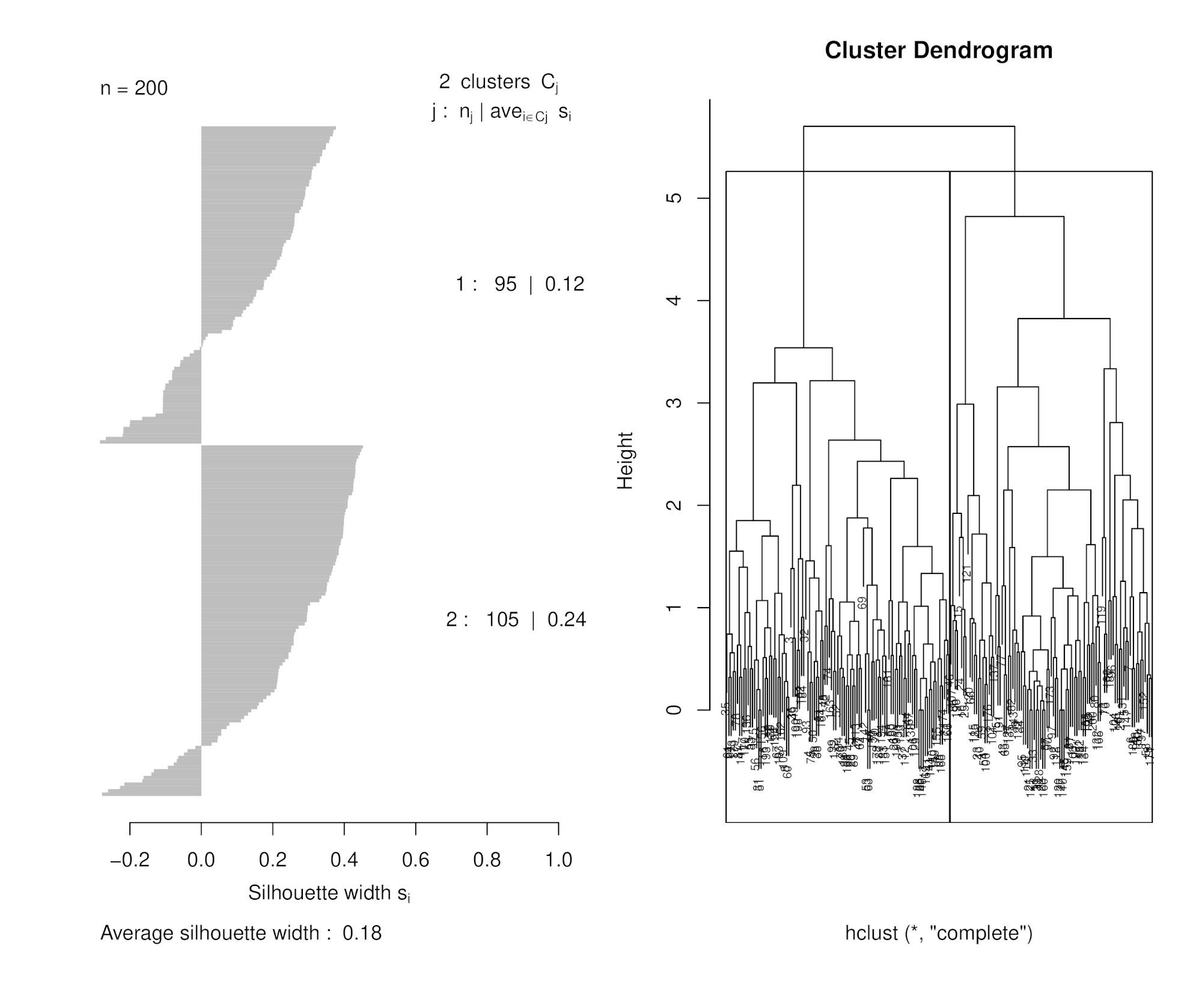

Supplement: S5 Fig — Silhouette width plot for the HAC clustering with 2 clusters (left) and relative dendrogram based on a complete linkage (right). The two selected clusters are highlighted inside the boxes. (TIFF) [file pone.0269584.s005.tiff]
