# Supplementary material for: Entia Non Sunt Multiplicanda … Shall I look for clusters in my cognitive data?
Source: PLoS One. 2022 Jun 30;17(6):e0269584. doi: 10.1371/journal.pone.0269584 (PMC9246139; doi:10.1371/journal.pone.0269584)
Supplement: S1 Table — Number of times of correct discover of a unique cluster (K = 1) over 500 replications and Average Rand Index (Mean and SD) by sample size (N), average correlation (r; null = 0, small = 0.2, large = 0.5), number of indicators, and clustering algorithm (Model-based Gaussian Clustering (MGC), Partitioning Around Medoids (PAM), Hierarchical Agglomerative Clustering (HAC)). (DOCX) [file pone.0269584.s006.docx]

**S1 Table. Clustering performance, one cluster/latent class.** Number of times of correct discover of a unique cluster (K=1) over 500 replications and Average Rand Index (Mean and SD) by sample size (N), average correlation (r; null=0, small=0.2, large=0.5), number of indicators, and clustering algorithm (Model-based Gaussian Clustering (MGC), Partitioning Around Medoids (PAM), Hierarchical Agglomerative Clustering (HAC)).

|  |  | **3 indicators** | | | | | | **6 indicators** | | | | | | **12 indicators** | | | | | |
| --- | --- | --- | --- | --- | --- | --- | --- | --- | --- | --- | --- | --- | --- | --- | --- | --- | --- | --- | --- |
|  |  | **MGC** | | **PAM** | | **HAC** | | **MGC** | | **PAM** | | **HAC** | | **MGC** | | **PAM** | | **HAC** | |
| **N** | **r** | **K=1** | **M (SD)** | **K=1** | **M (SD)** | **K=1** | **M (SD)** | **K=1** | **M (SD)** | **K=1** | **M (SD)** | **K=1** | **M (SD)** | **K=1** | **M (SD)** | **K=1** | **M (SD)** | **K=1** | **M (SD)** |
| 50 | null | 479 | 0.99 (0.06) | 493 | 0.99 (0.06) | 499 | 1 (0.02) | 492 | 1 (0.02) | 497 | 1 (0.04) | 498 | 1 (0.03) | 493 | 1 (0.02) | 499 | 1 (0.02) | 498 | 1 (0.03) |
| 100 | null | 498 | 1 (0.02) | 499 | 1 (0.02) | 500 | 1 (0) | 499 | 1 (0.02) | 499 | 1 (0.02) | 500 | 1 (0) | 500 | 1 (0) | 500 | 1 (0) | 500 | 1 (0) |
| 250 | null | 497 | 1 (0.03) | 493 | 0.99 (0.06) | 500 | 1 (0) | 500 | 1 (0) | 499 | 1 (0.02) | 500 | 1 (0) | 500 | 1 (0) | 500 | 1 (0) | 500 | 1 (0) |
| 500 | null | 497 | 1 (0.02) | 487 | 0.99 (0.08) | 500 | 1 (0) | 496 | 1 (0.04) | 500 | 1 (0) | 500 | 1 (0) | 496 | 1 (0.04) | 500 | 1 (0) | 500 | 1 (0) |
| 1000 | null | 483 | 0.99 (0.08) | 467 | 0.96 (0.13) | 500 | 1 (0) | 477 | 0.98 (0.09) | 496 | 1 (0.04) | 500 | 1 (0) | 468 | 0.97 (0.11) | 500 | 1 (0) | 500 | 1 (0) |
| 2000 | null | 467 | 0.97 (0.1) | 371 | 0.85 (0.26) | 500 | 1 (0) | 401 | 0.91 (0.18) | 474 | 0.97 (0.11) | 500 | 1 (0) | 254 | 0.77 (0.25) | 499 | 1 (0.02) | 500 | 1 (0) |
| 50 | small | 424 | 0.94 (0.14) | 448 | 0.95 (0.16) | 481 | 0.98 (0.09) | 233 | 0.77 (0.23) | 352 | 0.86 (0.22) | 400 | 0.91 (0.19) | 78 | 0.62 (0.18) | 225 | 0.74 (0.24) | 221 | 0.75 (0.23) |
| 100 | small | 379 | 0.9 (0.18) | 395 | 0.9 (0.2) | 469 | 0.97 (0.12) | 80 | 0.63 (0.18) | 242 | 0.75 (0.24) | 375 | 0.89 (0.2) | 2 | 0.53 (0.06) | 123 | 0.65 (0.21) | 147 | 0.7 (0.21) |
| 250 | small | 297 | 0.82 (0.22) | 155 | 0.66 (0.23) | 461 | 0.96 (0.13) | 11 | 0.54 (0.08) | 50 | 0.57 (0.15) | 313 | 0.83 (0.22) | 0 | 0.49 (0.04) | 54 | 0.58 (0.15) | 86 | 0.64 (0.18) |
| 500 | small | 311 | 0.83 (0.23) | 26 | 0.53 (0.11) | 438 | 0.94 (0.16) | 112 | 0.62 (0.2) | 9 | 0.53 (0.07) | 256 | 0.78 (0.23) | 4 | 0.45 (0.07) | 24 | 0.55 (0.11) | 54 | 0.63 (0.15) |
| 1000 | small | 430 | 0.93 (0.16) | 1 | 0.5 (0.03) | 431 | 0.93 (0.17) | 406 | 0.91 (0.2) | 3 | 0.52 (0.04) | 207 | 0.74 (0.22) | 414 | 0.9 (0.22) | 5 | 0.53 (0.06) | 54 | 0.63 (0.15) |
| 2000 | small | 465 | 0.97 (0.12) | 0 | 0.5 (0) | 377 | 0.88 (0.21) | 497 | 1 (0.04) | 0 | 0.51 (0.02) | 201 | 0.73 (0.22) | 500 | 1 (0) | 2 | 0.53 (0.05) | 24 | 0.61 (0.12) |
| 50 | large | 343 | 0.87 (0.21) | 68 | 0.58 (0.17) | 219 | 0.74 (0.23) | 134 | 0.61 (0.25) | 4 | 0.53 (0.07) | 45 | 0.62 (0.15) | 3 | 0.39 (0.08) | 1 | 0.54 (0.06) | 3 | 0.6 (0.12) |
| 100 | large | 436 | 0.94 (0.15) | 4 | 0.52 (0.05) | 136 | 0.67 (0.21) | 372 | 0.85 (0.26) | 0 | 0.53 (0.04) | 29 | 0.62 (0.13) | 82 | 0.46 (0.24) | 0 | 0.53 (0.05) | 4 | 0.61 (0.12) |
| 250 | large | 496 | 1 (0.05) | 0 | 0.51 (0.02) | 83 | 0.64 (0.17) | 496 | 0.99 (0.06) | 0 | 0.52 (0.03) | 14 | 0.6 (0.11) | 493 | 0.99 (0.08) | 0 | 0.53 (0.03) | 4 | 0.61 (0.11) |
| 500 | large | 500 | 1 (0) | 0 | 0.51 (0.01) | 64 | 0.64 (0.16) | 500 | 1 (0) | 0 | 0.51 (0.02) | 15 | 0.62 (0.12) | 500 | 1 (0) | 0 | 0.52 (0.03) | 3 | 0.62 (0.11) |
| 1000 | large | 499 | 1 (0.02) | 0 | 0.5 (0.01) | 82 | 0.66 (0.17) | 500 | 1 (0) | 0 | 0.51 (0.02) | 8 | 0.62 (0.12) | 500 | 1 (0) | 0 | 0.52 (0.03) | 1 | 0.63 (0.11) |
| 2000 | large | 500 | 1 (0) | 0 | 0.5 (0) | 70 | 0.64 (0.16) | 500 | 1 (0) | 0 | 0.51 (0.01) | 11 | 0.62 (0.12) | 500 | 1 (0) | 0 | 0.51 (0.02) | 2 | 0.63 (0.12) |
