# Supplementary material for: Entia Non Sunt Multiplicanda … Shall I look for clusters in my cognitive data?
Source: PLoS One. 2022 Jun 30;17(6):e0269584. doi: 10.1371/journal.pone.0269584 (PMC9246139; doi:10.1371/journal.pone.0269584)
Supplement: S2 Table — Number of times of correct discover of a unique cluster (K = 2) over 500 replications with a Cohen d = 0.4, and Average Rand Index (Mean and SD) by sample size (N), average correlation (r; null = 0, small = 0.2, large = 0.5), number of indicators, and clustering algorithm (Model-based Gaussian Clustering (MGC), Partitioning Around Medoids (PAM), Hierarchical Agglomerative Clustering (HAC)). (DOCX) [file pone.0269584.s007.docx]

**S2 Table. Clustering performance, two cluster/latent class, small differences**. Number of times of correct discover of a unique cluster (K=2) over 500 replications with a Cohen d=0.4, and Average Rand Index (Mean and SD) by sample size (N), average correlation (r; null=0, small=0.2, large=0.5), number of indicators, and clustering algorithm (Model-based Gaussian Clustering (MGC), Partitioning Around Medoids (PAM), Hierarchical Agglomerative Clustering (HAC)).

|  |  | **3 indicators** | | | | | | **6 indicators** | | | | | | **12 indicators** | | | | | |
| --- | --- | --- | --- | --- | --- | --- | --- | --- | --- | --- | --- | --- | --- | --- | --- | --- | --- | --- | --- |
|  |  | **MGC** | | **PAM** | | **HAC** | | **MGC** | | **PAM** | | **HAC** | | **MGC** | | **PAM** | | **HAC** | |
| **N** | **r** | **K=2** | **M (SD)** | **K=2** | **M (SD)** | **K=2** | **M (SD)** | **K=2** | **M (SD)** | **K=2** | **M (SD)** | **K=2** | **M (SD)** | **K=2** | **M (SD)** | **K=2** | **M (SD)** | **K=2** | **M (SD)** |
| 50 | null | 21 | 0.5 (0.01) | 2 | 0.5 (0.01) | 1 | 0.5 (0.01) | 6 | 0.5 (0.01) | 6 | 0.5 (0.02) | 1 | 0.5 (0.01) | 6 | 0.49 (0.01) | 2 | 0.5 (0.01) | 2 | 0.5 (0.01) |
| 100 | null | 7 | 0.5 (0) | 2 | 0.5 (0) | 2 | 0.5 (0.01) | 4 | 0.5 (0.01) | 2 | 0.5 (0.01) | 0 | 0.5 (0) | 0 | 0.5 (0) | 0 | 0.5 (0) | 0 | 0.5 (0) |
| 250 | null | 4 | 0.5 (0) | 3 | 0.5 (0) | 0 | 0.5 (0) | 4 | 0.5 (0.01) | 1 | 0.5 (0) | 0 | 0.5 (0) | 19 | 0.5 (0.03) | 0 | 0.5 (0) | 0 | 0.5 (0) |
| 500 | null | 6 | 0.5 (0) | 16 | 0.5 (0.01) | 0 | 0.5 (0) | 28 | 0.5 (0.01) | 8 | 0.5 (0.01) | 0 | 0.5 (0) | 128 | 0.53 (0.06) | 1 | 0.5 (0.01) | 0 | 0.5 (0) |
| 1000 | null | 19 | 0.5 (0.01) | 38 | 0.5 (0.01) | 0 | 0.5 (0) | 123 | 0.51 (0.03) | 25 | 0.5 (0.02) | 0 | 0.5 (0) | 358 | 0.57 (0.05) | 11 | 0.5 (0.01) | 0 | 0.5 (0) |
| 2000 | null | 52 | 0.5 (0.01) | 138 | 0.51 (0.02) | 0 | 0.5 (0) | 295 | 0.53 (0.03) | 76 | 0.51 (0.02) | 0 | 0.5 (0) | 480 | 0.6 (0.04) | 42 | 0.51 (0.02) | 0 | 0.5 (0) |
| 50 | small | 75 | 0.5 (0.01) | 48 | 0.5 (0.01) | 14 | 0.5 (0.01) | 235 | 0.5 (0.02) | 117 | 0.5 (0.02) | 93 | 0.5 (0.02) | 411 | 0.51 (0.03) | 248 | 0.5 (0.02) | 252 | 0.5 (0.02) |
| 100 | small | 109 | 0.5 (0.01) | 103 | 0.5 (0.02) | 23 | 0.5 (0.01) | 408 | 0.51 (0.02) | 235 | 0.5 (0.02) | 109 | 0.5 (0.01) | 476 | 0.51 (0.02) | 339 | 0.51 (0.02) | 311 | 0.5 (0.02) |
| 250 | small | 212 | 0.5 (0.01) | 314 | 0.51 (0.01) | 29 | 0.5 (0.01) | 481 | 0.51 (0.01) | 430 | 0.51 (0.02) | 158 | 0.5 (0.01) | 386 | 0.51 (0.01) | 449 | 0.51 (0.02) | 394 | 0.5 (0.01) |
| 500 | small | 204 | 0.5 (0.01) | 467 | 0.51 (0.01) | 52 | 0.5 (0.01) | 397 | 0.51 (0.01) | 489 | 0.51 (0.01) | 195 | 0.5 (0.01) | 98 | 0.52 (0.02) | 472 | 0.51 (0.01) | 423 | 0.51 (0.01) |
| 1000 | small | 99 | 0.5 (0) | 496 | 0.51 (0.01) | 64 | 0.5 (0.01) | 116 | 0.5 (0.01) | 498 | 0.51 (0.01) | 262 | 0.5 (0.01) | 2 | 0.52 (0.03) | 485 | 0.51 (0.01) | 449 | 0.5 (0.01) |
| 2000 | small | 15 | 0.5 (0) | 498 | 0.51 (0.01) | 90 | 0.5 (0.01) | 2 | 0.5 (0) | 500 | 0.51 (0.01) | 283 | 0.5 (0.01) | 64 | 0.52 (0.06) | 488 | 0.51 (0.01) | 458 | 0.5 (0.01) |
| 50 | large | 160 | 0.5 (0.02) | 397 | 0.51 (0.02) | 245 | 0.5 (0.02) | 238 | 0.51 (0.02) | 487 | 0.51 (0.02) | 438 | 0.5 (0.02) | 89 | 0.51 (0.02) | 493 | 0.51 (0.03) | 490 | 0.5 (0.02) |
| 100 | large | 74 | 0.5 (0.01) | 491 | 0.51 (0.01) | 332 | 0.5 (0.01) | 56 | 0.5 (0.01) | 500 | 0.51 (0.01) | 473 | 0.5 (0.01) | 6 | 0.51 (0.02) | 500 | 0.5 (0.01) | 492 | 0.5 (0.01) |
| 250 | large | 3 | 0.5 (0) | 500 | 0.51 (0.01) | 392 | 0.51 (0.01) | 0 | 0.5 (0) | 500 | 0.5 (0.01) | 485 | 0.5 (0.01) | 56 | 0.52 (0.06) | 500 | 0.5 (0.01) | 495 | 0.5 (0.01) |
| 500 | large | 1 | 0.5 (0) | 500 | 0.51 (0.01) | 399 | 0.51 (0.01) | 0 | 0.5 (0) | 500 | 0.5 (0.01) | 482 | 0.5 (0.01) | 221 | 0.61 (0.12) | 500 | 0.5 (0.01) | 499 | 0.5 (0) |
| 1000 | large | 1 | 0.5 (0) | 500 | 0.51 (0.01) | 413 | 0.5 (0.01) | 11 | 0.5 (0.02) | 500 | 0.5 (0.01) | 482 | 0.5 (0.01) | 414 | 0.72 (0.1) | 500 | 0.5 (0.01) | 499 | 0.5 (0) |
| 2000 | large | 2 | 0.5 (0) | 500 | 0.51 (0.01) | 431 | 0.51 (0.01) | 62 | 0.52 (0.05) | 500 | 0.5 (0.01) | 491 | 0.5 (0.01) | 454 | 0.75 (0.06) | 500 | 0.5 (0) | 500 | 0.5 (0) |
