# Supplementary material for: Entia Non Sunt Multiplicanda … Shall I look for clusters in my cognitive data?
Source: PLoS One. 2022 Jun 30;17(6):e0269584. doi: 10.1371/journal.pone.0269584 (PMC9246139; doi:10.1371/journal.pone.0269584)
Supplement: S3 Table — Number of times of correct discover of a unique cluster (K = 2) over 500 replications with a Cohen d = 0.8, and Average Rand Index (Mean and SD) by sample size (N), correlation (r; null = 0, small = 0.2, large = 0.5), number of indicators, and clustering algorithm (Model-based Gaussian Clustering (MGC), Partitioning Around Medoids (PAM), Hierarchical Agglomerative Clustering (HAC)). (DOCX) [file pone.0269584.s008.docx]

**S3 Table. Clustering performance, two cluster/latent class, large differences**. Number of times of correct discover of a unique cluster (K=2) over 500 replications with a Cohen d=0.8, and Average Rand Index (Mean and SD) by sample size (N), correlation (r; null=0, small=0.2, large=0.5), number of indicators, and clustering algorithm (Model-based Gaussian Clustering (MGC), Partitioning Around Medoids (PAM), Hierarchical Agglomerative Clustering (HAC)).

|  |  | **3 indicators** | | | | | | **6 indicators** | | | | | | | **12 indicators** | | | | | | | |
| --- | --- | --- | --- | --- | --- | --- | --- | --- | --- | --- | --- | --- | --- | --- | --- | --- | --- | --- | --- | --- | --- | --- |
|  |  | **MGC** | | **PAM** | | **HAC** | | **MGC** | | **PAM** | | **HAC** | | | **MGC** | | | **PAM** | | | **HAC** | |
| **N** | **r** | **K=2** | **M (SD)** | **K=2** | **M (SD)** | **K=2** | **M (SD)** | **K=2** | **M (SD)** | **K=2** | **M (SD)** | **K=2** | **M (SD)** | **K=2** | | **M (SD)** | **K=2** | | **M (SD)** | **K=2** | | **M (SD)** |
| 50 | null | 39 | 0.5 (0.03) | 30 | 0.5 (0.04) | 10 | 0.5 (0.03) | 110 | 0.54 (0.09) | 63 | 0.52 (0.07) | 41 | 0.51 (0.06) | 292 | | 0.69 (0.17) | 209 | | 0.6 (0.13) | 179 | | 0.58 (0.12) |
| 100 | null | 52 | 0.51 (0.04) | 63 | 0.52 (0.05) | 10 | 0.5 (0.02) | 240 | 0.6 (0.11) | 129 | 0.55 (0.09) | 64 | 0.52 (0.06) | 477 | | 0.81 (0.09) | 294 | | 0.63 (0.12) | 272 | | 0.63 (0.13) |
| 250 | null | 164 | 0.54 (0.06) | 189 | 0.55 (0.07) | 14 | 0.5 (0.02) | 461 | 0.69 (0.07) | 349 | 0.62 (0.09) | 93 | 0.53 (0.07) | 500 | | 0.84 (0.04) | 427 | | 0.68 (0.1) | 343 | | 0.65 (0.11) |
| 500 | null | 300 | 0.57 (0.06) | 369 | 0.59 (0.06) | 18 | 0.5 (0.02) | 499 | 0.71 (0.04) | 452 | 0.66 (0.07) | 121 | 0.54 (0.07) | 500 | | 0.84 (0.03) | 450 | | 0.69 (0.09) | 387 | | 0.66 (0.1) |
| 1000 | null | 395 | 0.59 (0.05) | 458 | 0.62 (0.04) | 30 | 0.51 (0.03) | 500 | 0.72 (0.03) | 488 | 0.68 (0.05) | 152 | 0.54 (0.07) | 499 | | 0.85 (0.02) | 466 | | 0.71 (0.08) | 427 | | 0.68 (0.09) |
| 2000 | null | 408 | 0.6 (0.05) | 495 | 0.63 (0.02) | 42 | 0.51 (0.03) | 499 | 0.72 (0.03) | 499 | 0.69 (0.04) | 162 | 0.54 (0.06) | 496 | | 0.85 (0.02) | 481 | | 0.73 (0.07) | 441 | | 0.68 (0.08) |
| 50 | small | 99 | 0.51 (0.05) | 62 | 0.51 (0.04) | 37 | 0.5 (0.04) | 239 | 0.55 (0.08) | 143 | 0.53 (0.07) | 104 | 0.52 (0.06) | 324 | | 0.66 (0.13) | 309 | | 0.6 (0.12) | 261 | | 0.56 (0.1) |
| 100 | small | 110 | 0.52 (0.04) | 147 | 0.53 (0.05) | 38 | 0.5 (0.03) | 338 | 0.58 (0.08) | 262 | 0.56 (0.08) | 124 | 0.52 (0.06) | 152 | | 0.68 (0.08) | 411 | | 0.63 (0.11) | 289 | | 0.55 (0.08) |
| 250 | small | 115 | 0.52 (0.04) | 294 | 0.55 (0.06) | 63 | 0.51 (0.03) | 180 | 0.59 (0.07) | 415 | 0.59 (0.07) | 182 | 0.52 (0.04) | 4 | | 0.69 (0.03) | 443 | | 0.65 (0.1) | 373 | | 0.54 (0.06) |
| 500 | small | 76 | 0.51 (0.03) | 419 | 0.57 (0.05) | 71 | 0.51 (0.03) | 43 | 0.55 (0.07) | 451 | 0.61 (0.07) | 210 | 0.53 (0.05) | 3 | | 0.68 (0.03) | 457 | | 0.65 (0.1) | 395 | | 0.54 (0.06) |
| 1000 | small | 20 | 0.5 (0.02) | 461 | 0.58 (0.04) | 99 | 0.51 (0.03) | 82 | 0.55 (0.1) | 470 | 0.61 (0.07) | 254 | 0.52 (0.04) | 407 | | 0.84 (0.09) | 467 | | 0.67 (0.1) | 419 | | 0.54 (0.05) |
| 2000 | small | 22 | 0.51 (0.03) | 481 | 0.58 (0.04) | 102 | 0.51 (0.03) | 298 | 0.65 (0.12) | 474 | 0.61 (0.06) | 287 | 0.53 (0.04) | 493 | | 0.88 (0.03) | 468 | | 0.66 (0.1) | 452 | | 0.54 (0.04) |
| 50 | large | 129 | 0.51 (0.04) | 323 | 0.53 (0.05) | 207 | 0.52 (0.04) | 161 | 0.53 (0.07) | 437 | 0.54 (0.07) | 406 | 0.53 (0.05) | 50 | | 0.62 (0.08) | 432 | | 0.56 (0.08) | 465 | | 0.52 (0.05) |
| 100 | large | 52 | 0.5 (0.03) | 457 | 0.54 (0.04) | 306 | 0.52 (0.03) | 20 | 0.51 (0.06) | 475 | 0.54 (0.05) | 440 | 0.52 (0.04) | 51 | | 0.63 (0.12) | 466 | | 0.55 (0.06) | 488 | | 0.52 (0.03) |
| 250 | large | 5 | 0.5 (0.02) | 494 | 0.53 (0.03) | 364 | 0.52 (0.03) | 84 | 0.56 (0.13) | 496 | 0.53 (0.04) | 473 | 0.52 (0.03) | 334 | | 0.84 (0.17) | 488 | | 0.54 (0.04) | 488 | | 0.51 (0.02) |
| 500 | large | 31 | 0.51 (0.06) | 500 | 0.53 (0.03) | 368 | 0.52 (0.02) | 203 | 0.65 (0.17) | 497 | 0.53 (0.03) | 480 | 0.52 (0.02) | 455 | | 0.94 (0.05) | 490 | | 0.53 (0.04) | 496 | | 0.51 (0.02) |
| 1000 | large | 61 | 0.53 (0.08) | 500 | 0.53 (0.03) | 403 | 0.52 (0.03) | 357 | 0.77 (0.13) | 500 | 0.53 (0.03) | 482 | 0.52 (0.02) | 486 | | 0.95 (0.03) | 496 | | 0.53 (0.03) | 498 | | 0.51 (0.02) |
| 2000 | large | 142 | 0.57 (0.1) | 500 | 0.53 (0.03) | 413 | 0.52 (0.02) | 406 | 0.8 (0.08) | 500 | 0.53 (0.02) | 484 | 0.52 (0.02) | 486 | | 0.95 (0.04) | 500 | | 0.52 (0.02) | 499 | | 0.51 (0.02) |
