# Supplementary material for: Entia Non Sunt Multiplicanda … Shall I look for clusters in my cognitive data?
Source: PLoS One. 2022 Jun 30;17(6):e0269584. doi: 10.1371/journal.pone.0269584 (PMC9246139; doi:10.1371/journal.pone.0269584)
Supplement: S4 Table — Number of times of correct discover of a unique cluster (K = 2) over 500 replications with a Cohen d = 1.5, and Average Rand Index (Mean and SD) by sample size (N), correlation (r; null = 0,small = 0.2,large = 0.5), number of indicators, and clustering algorithm (Model-based Gaussian Clustering (MGC), Partitioning Around Medoids (PAM), Hierarchical Agglomerative Clustering (HAC)). (DOCX) [file pone.0269584.s009.docx]

**S4 Table. Clustering performance, two cluster/latent class, very large differences**. Number of times of correct discover of a unique cluster (K=2) over 500 replications with a Cohen d=1.5, and Average Rand Index (Mean and SD) by sample size (N), correlation (r; null=0,small=0.2,large=0.5), number of indicators, and clustering algorithm (Model-based Gaussian Clustering (MGC), Partitioning Around Medoids (PAM), Hierarchical Agglomerative Clustering (HAC)).

|  |  | **3 indicators** | | | | | | **6 indicators** | | | | | | **12 indicators** | | | | | |
| --- | --- | --- | --- | --- | --- | --- | --- | --- | --- | --- | --- | --- | --- | --- | --- | --- | --- | --- | --- |
|  |  | **MGC** | | **PAM** | | **HAC** | | **MGC** | | **PAM** | | **HAC** | | **MGC** | | **PAM** | | **HAC** | |
| **N** | **r** | **K=2** | **M (SD)** | **K=2** | **M (SD)** | **K=2** | **M (SD)** | **K=2** | **M (SD)** | **K=2** | **M (SD)** | **K=2** | **M (SD)** | **K=2** | **M (SD)** | **K=2** | **M (SD)** | **K=2** | **M (SD)** |
| 50 | null | 383 | 0.74 (0.15) | 402 | 0.76 (0.15) | 286 | 0.66 (0.16) | 497 | 0.92 (0.06) | 498 | 0.89 (0.08) | 469 | 0.85 (0.12) | 498 | 0.99 (0.02) | 500 | 0.96 (0.05) | 499 | 0.96 (0.05) |
| 100 | null | 471 | 0.79 (0.09) | 491 | 0.81 (0.08) | 334 | 0.67 (0.14) | 500 | 0.93 (0.04) | 500 | 0.9 (0.06) | 488 | 0.85 (0.1) | 500 | 0.99 (0.01) | 500 | 0.97 (0.04) | 500 | 0.97 (0.04) |
| 250 | null | 494 | 0.82 (0.06) | 500 | 0.81 (0.05) | 415 | 0.7 (0.11) | 500 | 0.94 (0.03) | 500 | 0.91 (0.04) | 496 | 0.86 (0.08) | 500 | 0.99 (0.01) | 500 | 0.97 (0.02) | 499 | 0.97 (0.04) |
| 500 | null | 500 | 0.82 (0.05) | 500 | 0.82 (0.05) | 427 | 0.7 (0.1) | 500 | 0.94 (0.03) | 500 | 0.92 (0.03) | 497 | 0.86 (0.07) | 500 | 0.99 (0.01) | 500 | 0.98 (0.02) | 500 | 0.97 (0.03) |
| 1000 | null | 498 | 0.82 (0.04) | 500 | 0.82 (0.05) | 434 | 0.69 (0.1) | 500 | 0.94 (0.02) | 500 | 0.92 (0.03) | 496 | 0.86 (0.06) | 499 | 0.99 (0.01) | 500 | 0.98 (0.02) | 500 | 0.97 (0.02) |
| 2000 | null | 497 | 0.82 (0.04) | 500 | 0.83 (0.04) | 443 | 0.7 (0.09) | 494 | 0.93 (0.02) | 500 | 0.93 (0.03) | 499 | 0.86 (0.06) | 487 | 0.99 (0.02) | 500 | 0.98 (0.01) | 500 | 0.97 (0.02) |
| 50 | small | 319 | 0.7 (0.17) | 398 | 0.75 (0.14) | 270 | 0.66 (0.16) | 405 | 0.9 (0.08) | 484 | 0.88 (0.09) | 426 | 0.82 (0.15) | 295 | 0.93 (0.08) | 499 | 0.96 (0.05) | 433 | 0.92 (0.13) |
| 100 | small | 357 | 0.73 (0.16) | 479 | 0.8 (0.1) | 321 | 0.67 (0.14) | 320 | 0.88 (0.08) | 500 | 0.9 (0.07) | 436 | 0.83 (0.15) | 94 | 0.84 (0.09) | 500 | 0.96 (0.05) | 424 | 0.91 (0.14) |
| 250 | small | 371 | 0.76 (0.14) | 500 | 0.82 (0.07) | 359 | 0.67 (0.12) | 142 | 0.81 (0.09) | 500 | 0.92 (0.05) | 430 | 0.81 (0.14) | 0 | 0.76 (0.02) | 500 | 0.97 (0.03) | 405 | 0.89 (0.15) |
| 500 | small | 369 | 0.77 (0.14) | 500 | 0.82 (0.06) | 384 | 0.68 (0.12) | 272 | 0.85 (0.11) | 500 | 0.91 (0.05) | 411 | 0.8 (0.15) | 44 | 0.77 (0.07) | 500 | 0.97 (0.03) | 380 | 0.87 (0.16) |
| 1000 | small | 453 | 0.82 (0.1) | 500 | 0.83 (0.05) | 402 | 0.68 (0.11) | 469 | 0.93 (0.07) | 500 | 0.92 (0.05) | 409 | 0.79 (0.15) | 490 | 0.99 (0.04) | 500 | 0.98 (0.02) | 338 | 0.85 (0.15) |
| 2000 | small | 479 | 0.82 (0.08) | 500 | 0.82 (0.05) | 382 | 0.67 (0.11) | 499 | 0.95 (0.03) | 500 | 0.93 (0.04) | 390 | 0.76 (0.16) | 500 | 1 (0) | 500 | 0.98 (0.02) | 314 | 0.81 (0.15) |
| 50 | large | 212 | 0.67 (0.2) | 345 | 0.73 (0.16) | 224 | 0.61 (0.15) | 272 | 0.85 (0.17) | 393 | 0.86 (0.11) | 217 | 0.68 (0.15) | 145 | 0.82 (0.11) | 435 | 0.93 (0.09) | 137 | 0.76 (0.13) |
| 100 | large | 268 | 0.73 (0.21) | 419 | 0.78 (0.12) | 240 | 0.61 (0.13) | 436 | 0.92 (0.15) | 463 | 0.89 (0.11) | 211 | 0.69 (0.12) | 422 | 0.96 (0.09) | 475 | 0.95 (0.07) | 121 | 0.75 (0.1) |
| 250 | large | 363 | 0.81 (0.19) | 464 | 0.8 (0.11) | 256 | 0.6 (0.11) | 480 | 0.96 (0.09) | 484 | 0.89 (0.1) | 193 | 0.68 (0.12) | 499 | 1 (0.02) | 498 | 0.96 (0.06) | 154 | 0.72 (0.1) |
| 500 | large | 396 | 0.83 (0.17) | 492 | 0.8 (0.1) | 266 | 0.59 (0.09) | 486 | 0.97 (0.08) | 499 | 0.9 (0.09) | 229 | 0.67 (0.11) | 500 | 1 (0) | 500 | 0.96 (0.07) | 191 | 0.69 (0.1) |
| 1000 | large | 417 | 0.84 (0.16) | 492 | 0.81 (0.1) | 272 | 0.59 (0.09) | 492 | 0.97 (0.06) | 499 | 0.9 (0.09) | 238 | 0.67 (0.1) | 500 | 1 (0) | 500 | 0.96 (0.06) | 203 | 0.69 (0.11) |
| 2000 | large | 451 | 0.85 (0.14) | 496 | 0.82 (0.1) | 272 | 0.59 (0.09) | 489 | 0.97 (0.06) | 499 | 0.91 (0.09) | 279 | 0.66 (0.11) | 493 | 1 (0.01) | 500 | 0.96 (0.06) | 256 | 0.66 (0.11) |
