# Supplementary material for: Entia Non Sunt Multiplicanda … Shall I look for clusters in my cognitive data?
Source: PLoS One. 2022 Jun 30;17(6):e0269584. doi: 10.1371/journal.pone.0269584 (PMC9246139; doi:10.1371/journal.pone.0269584)
Supplement: Attachment — Submitted filename: Response to reviewers R2.docx [file pone.0269584.s011.docx]

*Reviewer #1: The authors had solved the primary issues in my previous comments. There are two minor issues the editor would decide if the authors have to answer them.*

RESPONSE: We thank the reviewer for his positive comment. His suggestions in the previous round of revision were very useful and contributed to improve the manuscript.

*1. I appreciate the authors presented more summary about their collected papers. On the other hand, the latest title is "… Shall I look for clusters in my cognitive data?". Obviously no cognitive papers were collected in the authors' review. Authors would consider the key terms in the title or emphasize the constraints in this article.*

RESPONSE: We prefer keeping the title as it is because it is the result of the previous round of revision and a specific suggestion from Reviewer 2. Indeed, as emphasized throughout the paper, our aims, analyses, and results are focused on quantitative cognitive psychology data. However, we are open to edit it and remove “in my cognitive data” or change it with “in my quantitative psychological data” if the Editor beliefs that this is really the best choice.

*2. I agree the authors' clarifications. Indeed we require the published data to estimate the false positive rate (FA). We have known high FA is the primary consequence of QRPs. I am curious if this simulation framework could be useful to estimate a latent false positive rate when the questionable practices are frequently identified in a set of papers. I understand this work is beyond the scope of this study. Still I encourage the authors could describe the potential every researcher of clustering methods use this simulation framework to monitor their practices in data analysis.*

RESPONSE: This point is very interesting, and it has the potential to open further venue of research. We have added a comment on how future studies using a simulation approach could help elucidate the impact of questionable research practices on the results obtained from clustering methods (see Discussion).

*Reviewer #2: All comments have been addressed satisfactorily. I specifically appreciate the decision tree and the explanation of the methods using an existing dataset (thank you for providing the R code).*

RESPONSE: We thank the reviewer for this positive comment.

*One final note: "In the present study, we examined the conditions under which popular techniques for clustering individuals may plausibly work on cognitive data, with a special focus on cognitive research." --> "with a special focus on cognitive research" is redundant now.*

RESPONSE: This sentence has now been corrected. Thank you very much for the careful reading of our manuscript.
